# Advanced quantitative CT for coronary artery disease: integrating stenosis quantification, plaque quantification, and fractional flow reserve-CT

**DOI:** 10.1093/bjr/tqag124

**Published:** 2026-05-26

**Authors:** Samuel Mihalcioiu, Bethlehem Mengesha, Chen Abitbol, Fionn Coughlan, Jonathon Leipsic, Alexander Haenel

**Affiliations:** Department of Radiology, St. Paul’s Hospital & University of British Columbia, Vancouver, BC V6Z 1Y6, Canada; Department of Radiology, St. Paul’s Hospital & University of British Columbia, Vancouver, BC V6Z 1Y6, Canada; Department of Radiology, St. Paul’s Hospital & University of British Columbia, Vancouver, BC V6Z 1Y6, Canada; Department of Medical Imaging, Fiona Stanley Hospital, Murdoch, WA, 6150, Australia; Department of Radiology, St. Paul’s Hospital & University of British Columbia, Vancouver, BC V6Z 1Y6, Canada; Department of Radiology, St. Paul’s Hospital & University of British Columbia, Vancouver, BC V6Z 1Y6, Canada; Department of Medical Imaging, Royal Columbian Hospital, New Westminster, BC, V3L 3M2, Canada; Department of Radiology and Nuclear Medicine, University Hospital of Schleswig-Holstein, Campus Lübeck, Lübeck, 23538, Germany

## Abstract

Coronary artery disease (CAD) continues to be a leading cause of death globally. Radiological evaluation of CAD generally consists of stenosis detection by cardiac CT angiography (CCTA). However, this approach can be inefficient and subject to interobserver variability. In this review, we explore the newest developments related to CAD evaluation by CCTA, with an emphasis on plaque quantification. Artificial intelligence–based quantitative CT offers an efficient and reproducible method to analyze plaque biomarkers that support more refined risk stratification for major adverse cardiovascular events. Artificial intelligence–based coronary stenosis quantification can streamline workflow and improve consistency. The utilization of a photon-counting detector CT has been demonstrated to enhance spatial resolution, thereby allowing more precise coronary lumen analysis and artifact reduction. Fractional flow reserve-CT delivers a noninvasive physiologic assessment of stenotic lesions. Finally, we discuss the impact of these changes on risk stratification and guiding preventive therapy as well as possible future directions in this rapidly evolving field.

## Introduction

Coronary artery disease (CAD) continues to be a leading cause of death globally.^1^ Visual assessment of cardiac CT angiography (CCTA) remains the current standard of practice to help guide the clinician’s management, yet this approach may be limited by inefficiency and interobserver variability,^2^ potentially delaying optimal care.

The radiological evaluation of CAD is rapidly changing, with new research and the rise of artificial intelligence (AI) offering solutions to these limitations. Plaque quantification by CCTA provides imaging biomarkers that are highly prognostic for major adverse coronary events (MACE),^2^ and offers a more nuanced risk stratification than stenosis assessment alone. However, technical challenges including blooming, beam-hardening, and coronary motion artifacts, and the intensive manual labor required for plaque segmentation and characterization have historically limited clinical implementation.^3^ Recent advances in AI may help overcome these obstacles and allow more efficient, accurate, and reproducible analysis.^4^

In this review, we explore the emerging AI applications for plaque quantification, stenosis analysis, and functional assessment with fractional flow reserve-CT (FFR-CT) along with the benefits provided by photon-counting detector CT (PCD-CT) for coronary spatial resolution. We discuss the clinical ramifications of these improvements, specifically regarding risk stratification, preventive therapy, and patient management. Finally, we address the possible future directions of this field.

## Plaque quantification

Plaque quantification by CCTA is the noninvasive assessment of different characteristics of coronary artery plaque.

High-risk plaque (HRP) features on CCTA have been defined based on histopathological correlates and include: (1) low-attenuation plaque (LAP) with attenuation <30-60 HU, representing a lipid-rich necrotic core; (2) positive remodeling (remodeling index >1.1), indicating outward vessel expansion; (3) napkin-ring sign, characterized by a ring of higher attenuation surrounding a necrotic core; and (4) spotty calcification (<3 mm in diameter). These features correlate with thin-cap fibroatheromas and vulnerable plaques identified at autopsy.^5–7^ A detailed overview of commonly used visual and quantitative plaque metrics and their definitions is provided in Table 1.

**Table 1:** Quantitative and visual coronary plaque metrics commonly used in CT plaque analysis.

Metric	Definition (HU thresholds)	Unit/clinical relevance	Remarks/assessment method	Prognostic implications (HR)	Key references
Total plaque volume (TPV)	Sum of all voxels within the vessel wall classified as plaque (irrespective of composition).	mm³; overall atherosclerotic burden	Quantitative (AI/semiautomated)	1.56 (95% CI 1.25–1.97)	^11^ ^,^ ^13^ ^,^ ^16^ ^,^ ^35^
Noncalcified plaque volume (NCPV)	Voxels with mean attenuation 30-349 HU.	mm³; fibrous/fibro-fatty plaque	Quantitative (AI/semiautomated)	1.21 (95% CI 0.94–1.56)	^2^ ^,^ ^6^ ^,^ ^8^ ^,^ ^11^ ^,^ ^16^ ^,^ ^35^
Low-attenuation plaque volume (LAPV)	Voxels with attenuation <30 HU.	mm³; lipid-rich necrotic core	Quantitative (AI/semiautomated)	1.60 (95% CI 1.10–2.34) 4.65 (95% CI 2.06–10.5) if >4% LAP burden	^6^ ^,^ ^8^ ^,^ ^102^
Calcified plaque volume (CPV)	Voxels with attenuation ≥350 HU.	mm³; mature calcified plaque	Quantitative (AI/semiautomated)	1.42 (95% CI 0.90–2.25)	^6^ ^,^ ^8^ ^,^ ^16^ ^,^ ^103^
1K plaque volume	Voxels with attenuation ≥1000 HU (“super-dense” calcification).	mm³; highly dense calcium	Quantitative; sometimes reported separately within CPV to assess blooming or stent-related artifacts		^103^ ^,^ ^104^
Low-attenuation plaque ratio (LAP/TPV)	Ratio of low-attenuation plaque to total plaque volume.	%	AI-based compositional metric		^8^ ^,^ ^35^
Noncalcified plaque ratio (NCP/TPV)	Ratio of noncalcified plaque to total plaque volume.	%	AI-based compositional marker		^11^ ^,^ ^16^
Percent atheroma volume (%AV)	Total plaque volume normalized to vessel or segment volume.	%	Longitudinal AI and serial CT studies	HR 4.37 (95% CI 2.51–7.62)	^11^ ^,^ ^13^ ^,^ ^16^
Remodeling Index (RI)	Ratio of vessel area at the lesion site to reference segment; RI > 1.1 = positive remodeling.	Ratio	Traditionally assessed visually or manually; can now be derived automatically in AI tools	HR 1.69; 95% (CI: 0.57–5.04)	^6^ ^,^ ^11^ ^,^ ^14^
Spotty calcification (SC)	Small (≤3 mm) calcific focus within plaque.	Presence/volume	Primarily a visual feature on manual review; some AI systems detect automatically	HR 2.2; 95% (CI: 1.1–4.3)	^6^ ^,^ ^14^ ^,^ ^102^
Napkin-ring sign (NRS)	Low-attenuation core surrounded by a high-attenuation rim on CCTA.	Binary (yes/no)	Morphological sign assessed visually; not inherently quantitative, though AI models can flag NRS-like features	HR 3.85; 95% (CI: 1.7–8.6)	^6^ ^,^ ^14^ ^,^ ^102^

Abbreviations: AI = artificial intelligence; CCTA = cardiac CT angiography.

### Prognostic evidence from prospective cohort studies

The prognostic value of CCTA-quantified atherosclerotic burden has been validated across multiple large-scale registries. The Scottish Computed Tomography of the Heart (SCOT-HEART) trial demonstrated that LAP burden >4% conferred an HR of 4.65 (95% CI, 2.06-10.5) for myocardial infarction (MI) or cardiac death, while the presence of any adverse plaque feature yielded an HR of 3.01 (95% CI, 1.61-5.63) over a 4.7-year follow-up.^8^^,^^9^ Extended 10-year follow-up confirmed sustained benefit, with CCTA-guided management reducing coronary heart disease death or non-fatal MI (6.6% vs 8.2%, HR 0.79, 95% CI, 0.63-0.99) alongside persistent increases in preventive therapy use.^10^ The Prospective Multicenter Imaging Study for Evaluation of Chest Pain (PROMISE) trial (*n* = 4415) revealed that HRP features in non-obstructive disease predicted events with HR 4.31 (95% CI, 2.25-8.26), maintaining independent prognostic value (adjusted HR 1.72, 95% CI, 1.13-2.62) after accounting for stenosis severity and clinical risk factors.^11^ In the Coronary CT Angiography Evaluation for Clinical Outcomes: An International Multicenter (CONFIRM) registry (*n* = 3547 patients without obstructive CAD) segment involvement score >5 predicted major adverse cardiac events with adjusted HR 3.3 (95% CI, 2.2-4.8), substantially exceeding the prognostic value of diabetes (HR 1.6, 95% CI, 1.2-2.1) or hypertension (HR 1.3, 95% CI, 1.0-1.6) over a 5.4-year follow-up.^12^ The International Study of Comparative Health Effectiveness with Medical and Invasive Approaches (ISCHEMIA) trial demonstrated that percent atheroma volume (PAV) >7.5% predicted major cardiovascular events with HR 4.37 (95% CI, 2.51-7.62).^13^ The Incident Coronary Syndromes Identified by Computed Tomography (ICONIC) study confirmed that the napkin-ring sign and LAP <60 HU represent powerful independent predictors of adverse outcomes.^14^ Building on these findings, the ongoing SCOT-HEART 2 trial (*n* > 5000) represents the first large-scale randomized evaluation of CCTA-guided versus risk score-guided primary prevention in asymptomatic individuals, testing whether subclinical atherosclerosis detection can enhance cardiovascular risk stratification and guide preventive therapy initiation.^15^

### Methodological approaches to plaque assessment

Three approaches have evolved for coronary plaque characterization, each with specific advantages and inherent limitations.^16–18^ Understanding these distinctions is essential for interpreting the existing literature and appreciating the evolution toward automated quantification.

Visual assessment by experienced observers remains the predominant clinical approach, employing qualitative or semiquantitative interpretation to identify plaque presence, classify composition (calcified, noncalcified, or mixed), and recognize high-risk morphological features.^17^ Semiquantitative scoring systems including segment involvement score (number of segments containing any plaque) and segment stenosis score (incorporating stenosis severity) provide reproducible estimates of disease extent. However, visual assessment suffers from substantial interobserver variability. Prospective multicenter studies demonstrate that stenosis severity is misclassified in 41% of cases even among experienced, certified readers.^19^^,^^20^ Agreement for HRP feature identification achieves only moderate concordance, with kappa statistics ranging from 0.56 to 0.69 across published validation studies.^21^

Quantitative plaque assessment employs systematic volumetric measurement through manual or semiautomated delineation of vessel wall and luminal boundaries on multiplanar reformatted images.^22–24^ Total plaque volume is derived as the difference between outer vessel wall volume and lumen volume, summed across the analyzed coronary segment. Composition analysis utilizes Hounsfield unit thresholds to partition total plaque volume into calcified, noncalcified, and low-attenuation components. PAV, calculated as plaque volume divided by vessel wall volume, normalizes burden to vessel size and demonstrates particular utility for serial assessment of therapeutic interventions. The landmark prognostic trials establishing plaque’s independent predictive value—SCOT-HEART, PROMISE, CONFIRM, and ISCHEMIA—uniformly employed these manual or semiautomated quantitative techniques.^8^^,^^11–13^ Analysis time ranges from 25 to 45 min per case, requiring specialized training and experience. Even experienced analysts demonstrate interobserver variability with coefficients of variation between 8% and 15% for volumetric measurements, while correlation with intravascular ultrasound (IVUS) typically ranges from *r* = 0.85 to 0.92.^22–24^

Artificial intelligence-enabled quantitative assessment leverages deep learning architectures to automate coronary plaque quantification, potentially enhancing reproducibility while dramatically reducing analysis time.^3^^,^^18^^,^^25^^,^^26^ Multicenter validation investigations demonstrate that state-of-the-art deep learning platforms achieve diagnostic accuracy between 81% and 86% compared to expert consensus reading, with correlation coefficients ranging from 0.92 to 0.95 when validated against IVUS.^3^^,^^18^^,^^26^ Analysis time decreases from over 25 min to typically <60 s per case.^3^ However, substantial platform-to-platform variability exists in algorithmic architecture, training dataset composition, and specific analytical capabilities.

Multiple commercial platforms are now available, offering different analytic approaches, levels of automation, and validation strategies, which are summarized in Table 2. For instance, HeartFlow Plaque Analysis^®^ (HeartFlow, Inc., Mountain View, California, United States) has been validated against IVUS,^26^ demonstrating high accuracy for both plaque volume and stenosis assessment. PlaqueIQ (Elucid Bioimaging, Inc., Boston, MA, United States) is validated against histology, providing detailed characterization of plaque composition.^27^ Cleerly Plaque Analysis^®^ (Cleerly, Inc., Denver, CO, United States) is supported by multicenter cohort studies.^28^^,^^29^ An exemplary AI-based quantitative CT (AI-QCT) analysis is illustrated in Figure 1.

**Figure 1:**
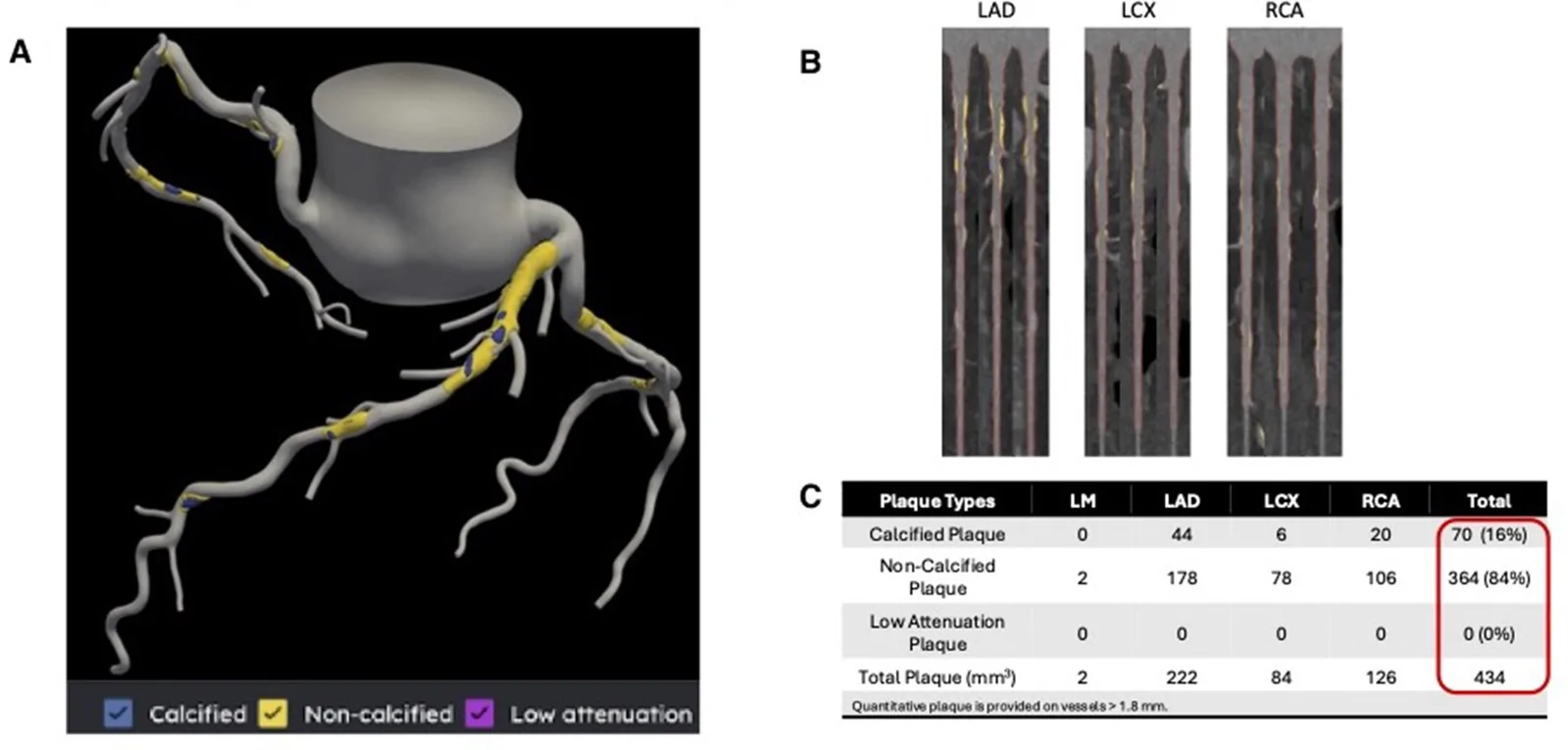
A 70-year-old male with vague pain underwent CCTA with AIQCPA. Total plaque volume of 434 mm^3^ strikingly noncalcified (yellow) for a patient of his age. (A) Interactive 3D map showing different types of plaque, mostly noncalcified. (B) MPR images of each vessel with automated segmentation of the lumen and plaque. (C) Quantified plaque volume breakdown by different components. All analysis performed using HeartFlow Plaque Analysis^®^ (HeartFlow, Inc.). Abbreviations: AI QCPA = artificial intelligence quantitative coronary plaque analysis, CCTA = cardiac CT angiography, MPR = multiplanar reconstruction.

**Table 2:** Commonly used commercially available AI-enabled coronary plaque and stenosis quantification software.

Vendor/product	Core analytical method	Plaque metrics reported	Stenosis quantification	Regulatory status (2025)	Notable strengths	Limitations	Sources
Circle cvi42 (Circle Cardiovascular Imaging, Canada)	AI-assisted segmentation	Plaque burden, volume, composition, remodeling index	Yes	FDA 510(k) cleared	PACS integration;		Circle Cardiovascular Imaging
Cleerly Plaque Analysis (Cleerly Health, USA)	Deep learning vessel and plaque segmentation	Calcified, noncalcified, and low-attenuation plaque; per-vessel volume and burden	Yes	FDA 510(k) cleared	Fully automated; validated in multicenter cohorts; longitudinal tracking		Cleerly Health
Elucid PlaqueIQ™ (Elucid, USA)	Biophysical tissue modeling trained on histopathology	Lipid-rich necrotic core, fibrous, and calcified plaque	Yes	FDA 510(k) cleared (2024)	Histologically validated	Computationally intensive	Elucid
HeartFlow Plaque Analysis (HeartFlow, USA)	AI-based plaque quantification integrated with CFD	Total and component plaque volume, stenosis metrics	Yes	FDA 510(k) cleared (2025)	Validated against IVUS; assessed in DECIDE trial; integrates with FFR-CT		HeartFlow
Medis Suite CT (Medis Medical Imaging, Netherlands)	Semiautomated segmentation and threshold quantification	Plaque burden, remodeling index, component volumes	Yes	CE marked; research use in USA	Widely used in research	Limited automation	Medis Medical Imaging
TeraRecon CT Cardiac (TeraRecon, USA)	Semiautomated quantitative and visual analysis	Plaque and stenosis evaluation	Yes	FDA cleared (part of AV platform)	Broad CT integration	Operator input required	TeraRecon
United Imaging Intelligence uAI Discover Coronary Plaque (United Imaging, China)	AI-based lesion and plaque detection	Plaque presence, burden, stenosis metrics	Yes	CE MDR Class IIa	Integrated with UIH CT scanners	Limited published validation	United Imaging
Caristo CaRi-Plaque™ (Caristo Diagnostics, United Kingdom)	AI-assisted plaque and stenosis quantification	Total plaque volume, calcified plaque volume, noncalcified plaque volume, low-density noncalcified plaque volume, plaque burden, remodeling index, maximum stenosis	Yes	FDA 510(k) cleared (2025); CPT code effective Jan 2026	Web-based platform; validated against SCCT levels 2 and 3 readers; seamless CCTA workflow integration; supports early CAD detection		Caristo Diagnostics
Siemens Healthineers (syngo.CT Coronary Analysis/syngo.via/syngo.via Frontier)	Automatic and AI-assisted coronary segmentation with advanced visualization; research applications via OpenApps	Coronary plaque burden, volume, composition, remodeling index; qualitative and quantitative plaque analysis	Yes	CE marked; FDA cleared for clinical products; research use only for syngo.via Frontier	Integrates with multiple CT vendors; OpenApps for advanced plaque and stenosis analyses; fast CAD rule-out; frontier offers research-grade advanced plaque phenotyping		Siemens Healthineers—syngo. CT Coronary Analysis, syngo.via, Frontier
APQ Health Autoplaque	Semiautomated AI-assisted	TPV, PAV, NCP, CP, LAP, HRP features	Yes (diameter stenosis)	FDA 510(k) cleared	Validated against IVUS; prognostic staging system		https://apqhealth.com/
Keya Medical DeepVessel FFR & Plaque (Keya Medical, USA/China)	Deep learning-based vessel segmentation with FFR-CT and plaque quantification	Total plaque volume, calcified, noncalcified, and mixed plaque; plaque burden; vessel volume	Yes (diameter stenosis)	FFR: FDA 510(k) cleared (2022), CE marked, NMPA approved, HSA certified; Plaque: Research use only (investigational device, not FDA cleared)	FFR validated in ADAPT study; fast processing; web-based platform with analyst-editable segmentations	Plaque not available for commercial sale in USA	https://www.keyamedical.com/deepvessel-ffr-ct/, https://www.keyamedical.com/deepvessel-plaque/

Abbreviations: ADAPT = assessment of the diagnostic performance of DeepVessel FFR in suspected coronary artery disease; AI = artificial intelligence; AV = atheroma volume; CAD = coronary artery disease; CCTA = cardiac CT angiography; CFD = computational fluid dynamics; CP = calcified plaque; DECIDE = artificial intelligence-derived plaque quantification: CCTA and AI-QCPA for determining effective CAD management; FFR-CT = fractional flow reserve-CT; HRP = high-risk plaque; HSA = health sciences authority; IVUS = intravascular ultrasound; LAP = low-attenuation plaque; NCP = noncalcified plaque; NMPA = national medical products administration; PACS = picture archiving and communication system; PAV = percent atheroma volume; SCCT = society of cardiovascular computed tomography; TPV = total plaque volume; UIH = united imaging healthcare.

Importantly, different manufacturers employ varying Hounsfield unit threshold schemes for plaque classification. Noncalcified plaque (NCP) is commonly defined as 30-350 HU, but low-attenuation component classification ranges from below 30 HU to below 60 HU depending on the specific algorithm, while calcified plaque thresholds range from above 130 HU to above 350 HU.^16^^,^^30^ The 2024 SCCT expert consensus document emphasized that these threshold variations introduce inter-platform differences in reported plaque volumes of 20%-30%, representing a significant barrier to generalizability and clinical adoption.^16^ Acknowledging these limitations is particularly important for serial CCTA imaging studies that investigate changes in atherosclerotic disease over time.^16^ According to Schulze, the limits of agreement for plaque quantification are quite wide between the baseline and follow-up study (+50% for total plaque volume [TPV]) and +100% for LAP).^2^ This variation can be attributed not only to differences in thresholds and software validation but also to scan parameters such as contrast concentration and tube voltage (kV), which can alter plaque attenuation.^16^ The identification of LAP depends on a fixed plaque attenuation cutoff of 30 HU, and any increase in the contrast concentration or decrease in kV will increase the overall plaque attenuation, thus giving a falsely lowered measurement of LAP.^16^ This is especially important in patients undergoing serial scanning to evaluate the therapeutic effect of certain medications by measuring the levels of plaque. If the scanning protocol and quantification software used are not consistent for follow-up exams, the measured plaque attenuation may be difficult to compare.^16^ The 2025 ACC expert consensus document recommends producing a vendor report for each exam, specifying the different parameters in order to improve standardization for serial imaging.^31^

#### Per-plaque vs per-vessel quantification

In addition to differences in methodology, an important distinction is the degree of detail of the analysis, whether focusing on a single plaque, whole vessel or whole-heart analysis.

The optimal approach depends on the clinical question being addressed.^24^^,^^32^^,^^33^

Per-plaque assessment characterizes individual lesions in detail—measuring length, volume, composition, remodeling index and high-risk features—which proves valuable for evaluating specific lesions considered for revascularization or correlating imaging with invasive FFR. Disadvantages include that focusing solely on discrete plaques misses diffuse atherosclerosis between obvious lesions.^32^^,^^33^

Per-vessel quantification measures total burden across entire coronary segments or the complete tree, generating metrics such as TPV, PAV, and segment involvement score.^24^^,^^33^ This approach captures the overall disease more efficiently and correlates better with cardiovascular outcomes.^33^ Major trials including SCOT-HEART, PROMISE, CONFIRM, and ISCHEMIA demonstrated that whole-heart metrics predict events independent of stenosis severity.^8^^,^^11^^,^^34^^,^^35^ The limitation is the potential difficulty in identifying focal high-risk features buried within extensive plaque.^33^

Current evidence favors per-vessel metrics for risk stratification and preventive therapy decisions.^8^^,^^11^^,^^34^^,^^35^ Per-plaque assessment remains valuable for characterizing specific lesions being considered for intervention or investigating culprit lesions in acute coronary syndromes (ACS) or to enhance intervention planning (Figure 1).^14^ In the future, hybrid approaches that combine both strategies may become more readily available, as the new machine learning-based approaches discussed in this review may allow these complex data to be processed in a short amount of time.^16^

#### Reference standard validation

The absence of a universally accepted reference standard complicates plaque validation and cross-platform comparability.^16^^,^^36^ IVUS provides deep tissue penetration for total plaque burden but limited spatial resolution (100-150 μm), insufficient for thin fibrous cap identification.^36^ Optical coherence tomography offers superior resolution (10-20 μm) but limited penetration depth (1-2 mm), precluding comprehensive plaque assessment.^36^ Combined IVUS-optical coherence tomography achieves only 69% positive predictive value for thin-cap fibroatheroma when validated against histopathology—substantially superior to either modality alone (41% for IVUS, 19% for optical coherence tomography) yet far from definitive.^36^^,^^37^ Moreover, intravascular imaging is restricted to patients undergoing catheterization, introducing selection bias that limits validation to a clinically restricted disease subset.^36^

Histopathology provides definitive plaque composition assessment, but sample sizes remain limited, tissue processing introduces artifacts, and the absence of pulsatile flow may alter plaque morphology.^37^^,^^38^ Expert consensus interpretation demonstrates inherent circularity—readers themselves show coefficients of variation 8%-15% for plaque volumes and only moderate agreement (*κ* = 0.4-0.6) for high-risk features, precluding validation against truly independent standards.^3^^,^^35^ Clinical outcomes represent ultimate validation, though requiring years of follow-up and providing no mechanistic insight at the individual plaque level.^13^^,^^35^^,^^39^

Current consensus supports multimodal validation: intravascular imaging for quantitative accuracy in high-grade lesions, histopathology for definitive composition when available, expert reading for practical quality control, and long-term outcomes for prognostic value.^16^^,^^36^^,^^37^ No single validation approach proves uniformly superior.^16^

#### Unresolved questions and future research directions

Despite accumulating evidence, the optimal plaque metric for cardiovascular risk prediction remains unestablished. Recent investigations employing automated quantification platforms provide insights into comparative performance across multiple parameters. A systematic review and meta-analysis by Bell and colleagues examining plaque quantification from CCTA demonstrated that quantitative plaque assessment significantly improved risk prediction beyond stenosis severity alone.^39^ AI-guided quantitative plaque staging in the ISCHEMIA cohort (*n* = 3711) showed that PAV exceeding 7.5% conferred HR 4.37 (95% CI, 2.51-7.62, *P* < .001), while each 559 mm^3^ increase in TPV yielded HR 1.56 (95% CI, 1.25-1.97, *P* = .001).^13^ Plaque volume metrics significantly improved discriminatory power at 6 months (AUC 0.688 vs 0.637, *P* = .006), 2 years (AUC 0.660 vs 0.617, *P* = .003), and 4 years (AUC 0.654 vs 0.608, *P* = .002) compared to clinical variables alone.^13^ Deep learning–enabled multicenter plaque quantification by Lin and colleagues (*n* = 1196) demonstrated that automated assessment achieved diagnostic accuracy of 84% versus expert readers and correlation *r* = 0.94 with IVUS, while reducing analysis time from 28 min to 45 s.^3^ Miller and colleagues reported patient-specific MI risk thresholds using AI-enabled plaque analysis, with HRs varying by plaque burden tertile.^40^ Dahdal and colleagues demonstrated that CCTA-derived plaque burden quantification offered enhanced prognostic value over coronary artery calcium (CAC) scoring in suspected CAD patients.^41^ The Bell meta-analysis pooled data across multiple studies, confirming that quantitative plaque parameters—particularly LAP and TPV—provided incremental prognostic value beyond traditional risk factors and stenosis severity.^39^ However, optimal metric selection likely differs by clinical context. Standardized risk stratification thresholds accounting for platform variability, scanner technology, and patient characteristics remain critical unmet needs for clinical implementation.^16^

In anticipation of the potential clinical implementation of AI-based plaque quantification, cardiovascular societies have begun releasing guidelines around its use. A 2025 ACC statement paper provides guidance on clinical indications, reporting templates, and standardization of AI-based plaque quantification.^31^

In addition, reimbursement pathways for AI-based plaque quantification are emerging but remain geographically restricted. Currently, only in the United States, automated plaque quantification and characterization from CCTA are recognized as reimbursable procedures.^42^ Outside North America, no dedicated reimbursement framework currently exists to our knowledge.

Wider clinical adoption will depend on analytical standardization, outcome validation, and demonstration of prognostic benefit and cost-effectiveness beyond stenosis-based assessment and traditional risk scores in large cohorts.

## Stenosis quantification

The CAD-RADS (Coronary Artery Disease Reporting and Data System) system has standardized the qualitative grading of stenosis.^4^^,^^43^ Stenosis grading by CAD-RADS provides reliable prognostic information regarding MACE, with higher grades shown to correlate with increased risk of MI.^43^ Accurate assessment of stenosis severity is critical because these results guide clinical management decisions, including referral for invasive coronary angiography (ICA) and revascularization.^44^^,^^45^ Visual assessment remains the current clinical standard for stenosis quantification. However, visual stenosis evaluation on CCTA is time-consuming and prone to significant interobserver variability.^46^ In the PROMISE trial, certified CCTA readers misclassified the severity of stenosis in 41% of cases, with overestimation being more common among less experienced readers.^46^

Similar to plaque quantification, AI-based coronary stenosis quantification (AI-CSQ) has emerged as a tool to improve efficiency and diagnostic accuracy. As shown in Table 2, the vast majority of commercially available software offers automated- or semiautomated stenosis detection and quantification as part of their workflow. These tools can provide reproducible measurements of percent diameter stenosis, minimal lumen area and cross-sectional luminal reduction by combining vessel contour detection with geometric and intensity-based measurements. AI-assisted stenosis quantification can contribute through various mechanisms at different levels of the analysis workflow: (1) automated centerline detection and vessel tracking, which facilitates manual or semiautomated lumen measurement; (2) lumen segmentation and stenosis quantification, providing diameter and area measurements; and (3) complete integrated assessment combining anatomy, stenosis severity, and hemodynamic significance. Different platforms offer different capabilities along this spectrum.

AI-CSQ shows a strong correlation with IVUS.^47^ Recent studies demonstrate that AI-CSQ outperforms level 2-certified CCTA readers across all clinical scenarios and even surpasses level 3-certified readers in patients with above-median plaque burden.^46^ This highlights its potential to assist with challenging cases, including those reviewed by expert readers. In addition to improving diagnostic accuracy, AI-CSQ could potentially help to triage CCTA studies by flagging cases with significant obstruction for urgent review.

AI-CSQ could offer advantages in efficiency and workflow optimization. Automated detection of stenosis could help to substantially reduce interpretation time, enabling radiologists to increase case throughput while maintaining consistency. By reducing interobserver variability, AI-CSQ also may have the potential to standardize reporting across institutions and improve inter-reader agreement for CAD-RADS classification.^48^

For successful clinical implementation, the robustness and generalizability of AI-CSQ must be ensured. Key challenges include understanding its performance across different CT acquisition parameters, scanner platforms, and diverse patient populations. Comparative analyses between vendors will also be necessary to establish reproducibility and ensure standardization before widespread adoption. The 2021 Society of Cardiovascular Computed Tomography expert consensus document emphasizes that automated tools should supplement, not replace, expert visual assessment, and all automated measurements require quality control review by experienced readers.^49^

## The role of plaque quantification in risk stratification and guiding preventive therapy

The 2019 ACC/AHA (American College of Cardiology/American Heart Association) Guideline on primary prevention of cardiovascular disease, the 2019 ESC/EAS (European Society of Cardiology/European Atherosclerosis Society) Guidelines for the management of dyslipidemias and the 2021 ESC Guidelines on cardiovascular disease prevention all recommend using risk scores such as PCE, ASCVD risk estimator, SCORE2 or SCORE-OP in identifying patients at higher risk for cardiovascular morbidity and mortality.^50–52^ These risk scores are based on traditional risk factors, including patients' age, sex, serum low-density lipoprotein (LDL) or high-density lipoprotein (HDL), and total cholesterol level, and the presence of hypertension, diabetes mellitus, and smoking history, and do not incorporate objective evidence of coronary atherosclerosis based on imaging. However, among patients with an intermediate-risk score, the guidelines recommend using risk modifiers such as the coronary calcium score.^50–52^ CAC score is a robust and well-validated imaging biomarker that has been shown to predict cardiovascular mortality even among individuals without traditional cardiovascular risk factors (CVRF).^53^ However, this method does not account for the presence of NCP and can underestimate risk among younger individuals with lower calcified plaque (CP) volume but higher NCP volume.^2^ Results from the SCOT-HEART trial have shown LAP burden to be the strongest predictor of future MI among patients with stable chest pain, irrespective of cardiovascular risk score, CAC score, and coronary artery luminal stenosis.^8^ Age- and gender-adjusted percentile curves based on quantification of TPV can provide a more comprehensive and individualized approach to risk stratification.^54^ A recent consensus statement by the quantitative cardiovascular imaging study group recommends pharmacologic treatment in the presence of any atherosclerotic plaque and high-intensity statin if TPV is in the 70th percentile.^2^ The recent 2025 American Heart Association statement refers to non-obstructive VAD as a heterogenous “non-benign” condition.^55^ The risk of its progression to MACE is based on the clinical factors and adverse plaque features identified on CCTA, with the AHA suggesting that preventive therapies should be based on individual risk profiles that incorporate CCTA.^55^

The effect of lipid-lowering therapy on plaque volume and composition has been studied using invasive imaging modalities such as IVUS or optical coherence tomography (OCT) and by using noninvasive means such as CCTA.^56^^,^^57^ Statin therapy is known to have pleiotropic effects on vascular health including improved endothelial function, reduction in vascular inflammation and oxidative stress, as well as stabilization of atherosclerotic plaque.^58^ Changes in plaque volume or a change of NCP to stable CP can represent plaque regression. A meta-analysis by Banach et al has shown significantly high-intensity statin therapy to result in a reduction of fibrous plaque volume along with a significant increase in dense calcium volume.^56^ Similarly, a meta-analysis including 51 studies and 9113 patients has shown a significant reduction in PAV and total atheroma volume associated with lipid-lowering therapy, which was mainly driven by high-intensity statin therapy.^57^ The Effect of Vascepa on Improving Coronary Atherosclerosis in People with High Triglycerides Taking Statin Therapy (EVAPORATE) trial has shown the reduction in low-attenuation NCP volume following 18 months of therapy with Icospent ethyl.^59^ In addition, the Global Assessment of Plaque Regression With a PCSK9 Antibody as Measured By Intravascular Ultrasound (GLAGOV) trial has demonstrated a significant reduction in TPV among patients on statin therapy who received the PCSK9 inhibitor evolocumab compared to placebo.^60^ An additional potential advantage of CCTA-guided risk stratification was revealed by a recent sub-study of the SCOT-HEART 2 trial. In comparison with conventional CVRF scoring, CCTA-guided risk stratification resulted in enhanced patient acceptance of recommended preventive therapies, improved risk factor modification, and better primary prevention targeting.^61^

Given that plaque stabilization under statin therapy increases the total volume of CP, the role of the CAC score among patients on statin therapy is of uncertain significance.^2^^,^^62^ Patients under primary prevention treatment strategies are typically monitored by using surrogates such as serum LDL level and percentage reduction of LDL rather than direct quantifiable changes that occur in the atherosclerotic plaque volume and composition under lipid-lowering therapy. A potential challenge for serial imaging is the variability in plaque attenuation resulting from different scan protocols, making comparison difficult.^16^ While a treatment strategy optimized using a more comprehensive approach incorporating plaque quantification along with clinical risk factors and serum biomarkers is favorable, the real-world application of plaque quantification in optimizing and titrating lipid-lowering therapy in the setting of primary prevention will need to be further studied.

## Plaque quantification using photon-counting detector CT

Photon-counting detector CT is a novel technology that provides superior spatial resolution and spectral post-processing capabilities compared to conventional multidetector CT.^63^ Unlike conventional multidetector CT scanners, which use energy-integrating detectors (EID), PCD-CT utilizes specialized semiconductor detectors with no septa that are made of silicon, cadmium zinc telluride, or cadmium telluride that directly convert individualized x-ray photons to electric signal.^63^^,^^64^ The high-resolution imaging coupled with the ability to characterize tissue components makes it ideal to assess atherosclerotic plaque volume and composition.

One of the commonly encountered challenges in assessing CAD using a standard multidetector CT includes blooming artifacts and beam-hardening artifacts that are commonly encountered in the presence of densely calcified coronary plaque or stents. Calcium blooming, which occurs due to partial volume averaging, can result in overestimation of luminal stenosis and plaque volume quantification. The high spatial resolution and spectral decomposition capability in PCD-CT can overcome such challenges by providing a more accurate means to quantify CP volume. The use of PCD-CT at ultra-high spatial resolution (UHR) has been shown to significantly reduce percentage diameter stenosis and improve diagnostic accuracy of coronary artery stenosis measurements when compared to EID-CT.^65^ This was largely driven by the reduction in percentage diameter stenosis in the presence of CP.^65^ Studies have reported a 49%-54% reclassification rate to lower CAD-RADS categories using UHR PCD-CT.^66^^,^^67^ The spectral capability of PCD-CT, which allows for calcium removal image reconstruction, was shown to significantly reduce blooming artifact and improve stenosis measurement accuracy.^67^ Furthermore, UHR PCD-CT improved the reproducibility of LAP quantification and significantly reduced TPV quantification by almost one-third compared to conventional multidetector CT.^66^ Given the differences in imaging resolution between EID-CT and PCD-CT, validation studies may be needed to redefine PCD-CT–specific detection thresholds of plaque components compared to either IVUS imaging or histology.^66^

Current SCCT recommendations for use of CCTA in symptomatic patients with prior coronary stent are mainly supported for patients with intracoronary stent diameters of ≥3.0 mm, where it is deemed appropriate, while its application in smaller stents (stent diameter <3.0 mm and thin strut <100 micrometers) receives a lower strength recommendation (may be appropriate).^49^ The enhanced spatial resolution, as well as the intrinsic capability of UHR PCD-CT to overcome blooming and beam-hardening artifact, enables better in-stent luminal visibility and improved diagnostic accuracy. The diagnostic performance of UHR PCD-CT in coronary stent patency has been studied and was shown to have excellent performance (sensitivity of 100%, specificity of 87%, accuracy of 89%, and negative predictive value of 100%) compared to ICA.^67^ In addition, improved stent structure visualization allows identification of stent-related complications such as stent malapposition and stent fracture.^67^ Given the groundbreaking advancement and enhanced capabilities of PCD-CT, the clinical utility of CCTA in coronary stent assessment will likely increase in the future.

## Fractional flow reserve-CT

Quantification of stenosis and plaque on CCTA provides essential anatomic and prognostic insights, but these measures alone cannot reliably determine whether stenoses are associated with abnormal physiology. This distinction is clinically critical, as studies show that only about half of stenoses identified on CCTA are functionally significant when assessed by invasive FFR.^68^ FFR, the reference standard for isolating lesion-specific functional significance, is measured during ICA and^69^ is defined as the ratio of mean coronary pressure distal to a stenosis to mean aortic pressure during maximal hyperemia. Clinically, an FFR ≤ 0.80 is widely accepted to indicate ischemia-causing lesions, but importantly, physiology is not a binary measure but rather a continuous variable with lower FFR values associated with worsened outcomes and deriving greater benefit from revascularization. By standardizing coronary resistance, FFR more accurately predicts the benefit of revascularization than anatomy alone, guiding treatment in patients with significant stenoses.^70^^,^^71^

Despite being the gold standard for functional assessment, the broad adoption of invasive FFR is limited by its invasive nature, high costs for pressure wires, and the need for adenosine administration.^72^ To overcome these limitations, FFR derived from CCTA (FFR-CT) was developed, combining noninvasive anatomical and functional assessments.

FFR-CT can be calculated using 2 distinct methodological approaches with important differences in computational requirements and workflow. Conventional computational fluid dynamics (CFD)-based methods (eg, HeartFlow, Mountainview, CA) construct a patient-specific 3D coronary model, which provides the framework for simulating blood flow.^73^ Applying the Navier-Stokes equations and assuming blood behaves as an incompressible Newtonian fluid, the model estimates pressure and flow at rest and during simulated hyperemia, eliminating the need for adenosine infusion. This enables clinicians to evaluate both the anatomic severity and physiologic impact of coronary lesions noninvasively, thereby enriching catheterization laboratory referral. These methods require off-site supercomputer analysis with typical turnaround times of several hours, though they provide comprehensive hemodynamic assessment with established accuracy.

Newer AI-based or machine learning-enhanced approaches utilize trained neural networks or reduced-order models to estimate FFR from anatomic features without full CFD simulation. These methods enable rapid on-site computation within minutes using standard workstations, facilitating immediate clinical decision-making. However, they typically require larger training datasets, and their accuracy may vary across different anatomic scenarios and degrees of stenosis. Both approaches show good correlation with invasive FFR (correlation coefficients 0.7-0.9), but direct head-to-head comparisons are limited. The choice between conventional CFD- and AI-based methods often reflects considerations of accuracy, speed, cost, and infrastructure requirement.

Validation of CFD-based FFR-CT as a noninvasive tool has been well established in multiple trials.^74–77^ While CCTA is highly sensitive, it lacks specificity and frequently overcalls intermediate (40-90%) stenoses. In the analysis of coronary blood flow using CT angiography: Next Steps (NXT) trial, FFR-CT significantly improved specificity (79% vs 34%) and overall diagnostic accuracy (AUC 0.90 vs 0.81) compared with CCTA, while maintaining high sensitivity.^76^ The Prospective Comparison of Cardiac PET/CT, SPECT/CT Perfusion Imaging, and CT Coronary Angiography With Invasive Coronary Angiography (PACIFIC) trial further confirmed these advantages, demonstrating higher diagnostic accuracy than both CCTA and functional imaging modalities such as SPECT and PET.^78^

Beyond diagnostic accuracy, randomized trials including the Prospective Randomized Trial of the Optimal Evaluation of Cardiac Symptoms and Revascularization (PRECISE) and the FFR derived from computed tomography coronary angiography in the assessment and management of stable chest pain (FORECAST) trial showed that FFR-CT reduces false positives and lowers the rate of unnecessary invasive angiography while improving catheter laboratory efficiency with similar clinical outcomes.^79^^,^^80^ The Assessing Diagnostic Value of Noninvasive FFRCT in Coronary Care (ADVANCE) registry showed that incorporating FFR-CT into routine care altered treatment strategy in two-thirds of patients,^81^ and those with normal FFR-CT values (>0.80) had markedly lower risks of cardiovascular death or MI than those with abnormal values (5.8% vs 38.4%). Beyond diagnosis, FFR-CT provides incremental prognostic information: abnormal values predict worse 1- and 3-year outcomes.^82^^,^^83^ Conversely, a normal lesion-specific FFR-CT was associated with very low annual event rates (<0.5%), supporting safe deferral of intervention. Reflecting this evidence, the 2021 ACC/AHA chest pain guidelines recommend FFR-CT for intermediate-risk patients with suspected CAD (class IIa), especially in 40-90% stenoses where physiologic assessment adds most value.^84^ Recently published data indicated that multicenter implementation of FFR-CT in the United Kingdom was safe, beneficial to the patient pathway, and led to reduced unnecessary downstream cardiac testing.^85^

All major validation trials mentioned above utilized conventional CFD-based FFR-CT (HeartFlow), requiring off-site analysis. Data for on-site AI-based FFR-CT approaches come primarily from smaller validation cohorts. The MACHINE consortium across 5 international centers established a diagnostic accuracy of 78% per vessel and 85% per patient (AUC 0.84).^86^ The Effect of On-Site CT-Derived Fractional Flow Reserve on the Management of Decision Making for Patients With Stable Chest Pain (TARGET Trial) in 1216 patients enrolled across six Chinese medical centers confirmed safe clinical implementation, with real-world studies demonstrating potential to reduce unnecessary invasive angiography by over 50%. Despite an overall increased revascularization, no improvement in symptoms or quality of life or reduction in MACE was observed.^87^ When interpreting FFR-CT literature, readers should note which computational method was employed, as accuracy, speed, and workflow implications differ between approaches.

Equivalent to invasive measures, the cutoff for FFR-CT is ≤0.80 to denote a hemodynamically significant lesion. Some authors consider a “gray zone” between ∼0.76 and 0.80, where interpretation of ΔFFR-CT (pressure drop across the stenosis) becomes important.^88^ Rather than using FFR-CT as a binary tool with a fixed cutoff point, it should be interpreted as a continuous variable. ΔFFR-CT reflects lesion-specific hemodynamic impact rather than cumulative downstream pressure loss assessed by a distal FFR-CT measurement. Distal FFR-CT values may be influenced by diffuse disease, downstream microvascular resistance, and/or serial lesions. Growing evidence supports the added value of ΔFFR-CT to access lesion-specific ischemia, improving specificity and guidance of revascularization.^89^ Optimized ΔFFR-CT cutoff values published in the literature are ranging between 0.06 and 0.13.^89–92^ These differences likely reflect variations in lesion morphology, vessel size and shape, imaging quality, computational modeling assumptions, and measurement locations. Figure 2 illustrates an exemplary case that highlights the additional value of ΔFFR-CT assessment.

**Figure 2:**
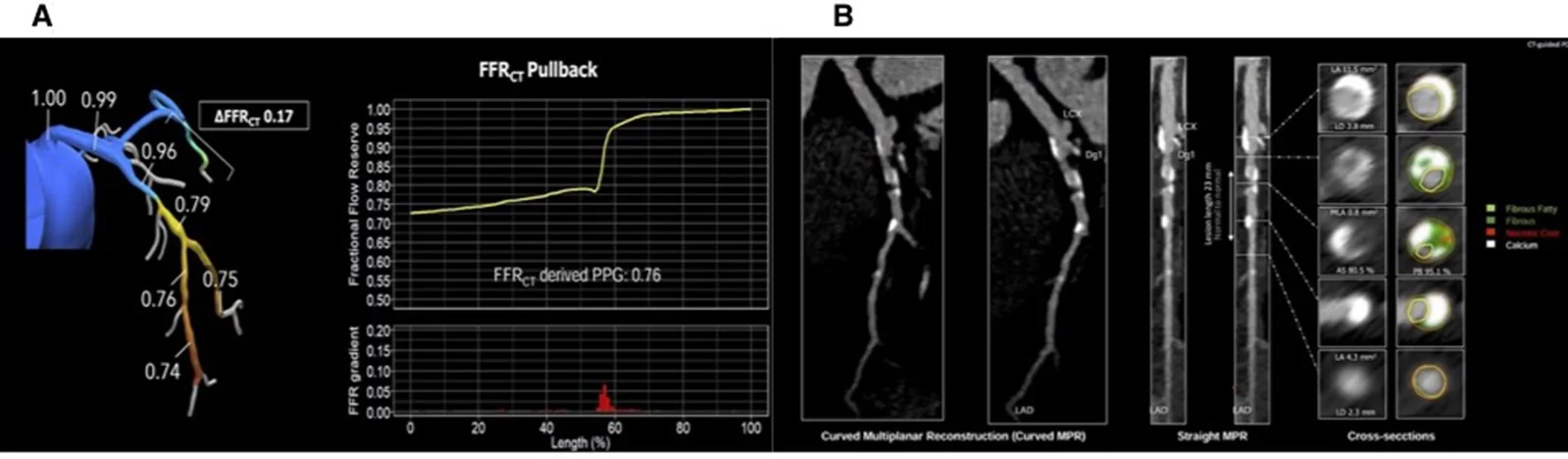
A 72-year-old with shortness of breath underwent CCTA identifying a 70-90% CCTA stenosis. Underwent FFRct analysis (A) which documented a gray zone value of 0.76 but a high delta FFRct (0.17) (B) with extensive calcified and noncalcified plaque noted. Analysis performed using HeartFlow FFR_CT_ Analysis (HeartFlow, Inc.). Abbreviations: CCTA = cardiac CT angiography, FFRct = fractional flow reserve derived from CT.

An increasing amount of evidence demonstrates that plaque characteristics are closely linked to ischemia. This supports the idea of integrating lesion-specific FFR-CT with quantitative plaque analysis. This comprehensive approach links the functional significance of a lesion with its anatomical and compositional features.^93^^,^^94^ Gaur et al demonstrated that integrating FFR‑CT with plaque features improves the sensitivity and specificity for identifying ischemia-prone lesions.^95^ Moreover, the combination of CCTA-derived plaque features with FFR‑CT enhanced MACE prediction compared with either measure alone (Figure 3).^96^ A recent secondary analysis of the ADVANCE Registry demonstrated that AI-enabled quantitative plaque volume independently predicts MACE, even after adjusting for distal FFR‑CT and ΔFFR‑CT, highlighting the incremental value of combining functional assessment and AI-enabled plaque quantification.^97^

**Figure 3:**
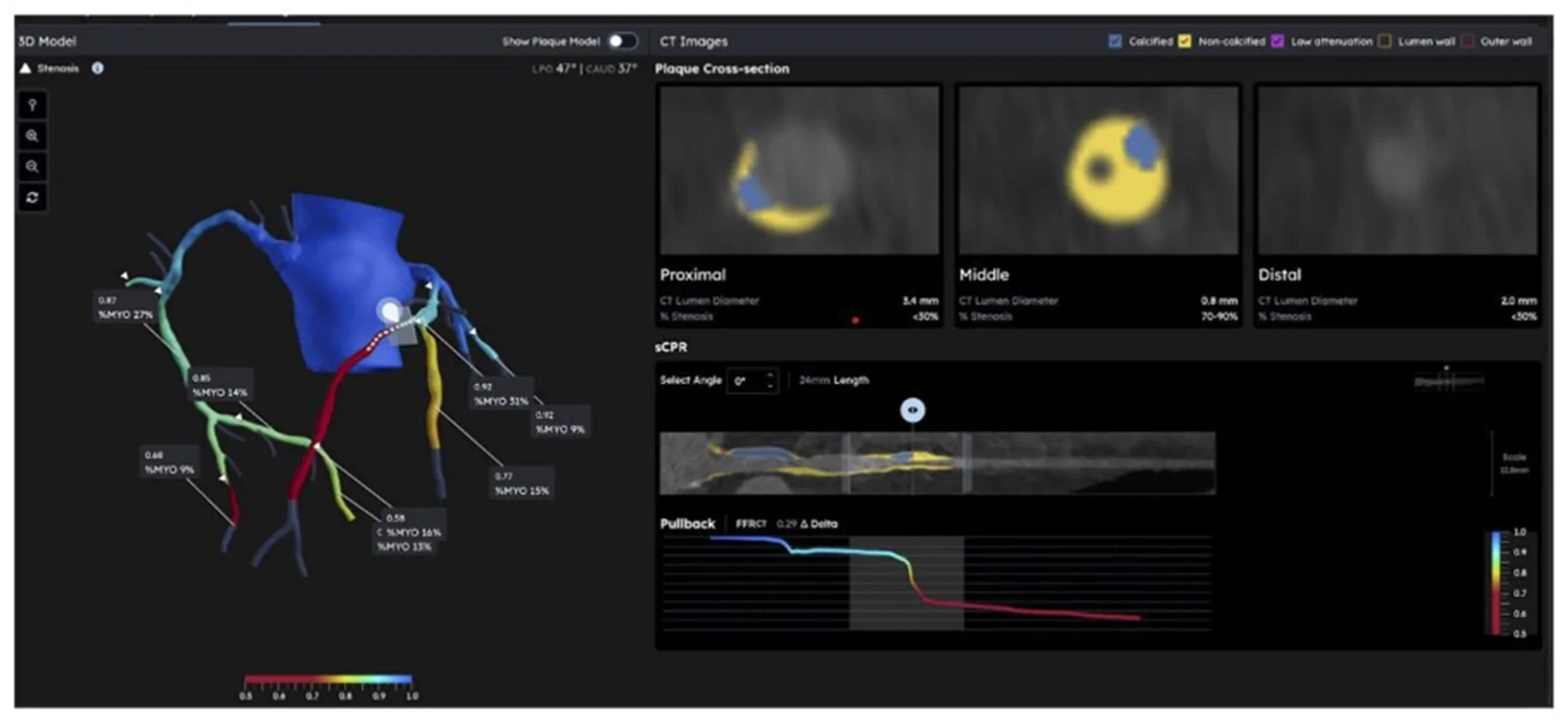
Interactive plaque and physiology interrogation in a patient referred for consideration of PCI (percutaneous coronary intervention). FFRct model displays a bifurcating lesion with a focal pattern of physiology with a high delta FFRct. The plaque at the stenosis is shown to be largely noncalcified (yellow). All analysis performed using HeartFlow FFR_CT_ Analysis And Plaque Analysis^®^ (HeartFlow, Inc.). Abbreviation: FFRct = fractional flow reserve derived from CT.

Another bioimaging marker, endothelial shear stress (ESS), measures the frictional force that blood exerts on endothelial cells.^98^ Endothelial shear stress is derived from CFD analysis of coronary geometry, calculating the tangential force per unit area exerted by blood flow on the vessel wall.^98^

Both high and low ESS levels have been shown to have strong prognostic values for ACS as they promote plaque development and destabilization.^98^ The measurement of ESS by CCTA has been shown to be similar to IVUS.^98^^,^^99^ While ESS is an important driver of the formation of atherosclerosis and plaque destabilization, the magnitude of forces associated with axial plaque forces is significantly greater. While axial plaque forces are not routinely measured or calculated, it has been shown that trans lesional pressure loss defined by delta FFR-CT is a surrogate measure of axial plaque forces. These calculated forces have been shown to be markers of adverse plaque features and also future plaque rupture and incident MI.^25^^,^^100^

Stenosis quantification identifies obstructive lesions, while quantitative plaque analysis provides highly relevant prognostic data; however, neither approach alone reliably predicts ischemia. FFR-CT complements both by establishing lesion-specific physiological significance, enabling the identification of patients and lesions most likely to benefit from revascularization. Integrating FFR-CT with plaque quantification, therefore, provides a more holistic understanding of CAD, improving risk stratification and supporting individualized management strategies.

Despite these advantages, several technical and clinical considerations influence the accuracy of FFR-CT. High-quality CCTA acquisition is essential, as poor contrast, motion, or calcium blooming can impair lumen modeling and flow simulation. Additional barriers include limited accessibility, the need for complex off-site computational analysis, and substantial costs, particularly outside the United States and United Kingdom.^101^

## Future directions

Although the accuracy of AI-QCT has been validated, its robustness and reproducibility must be further proven in large, multicenter trials to ensure that the AI algorithms produce consistent measurements and function effectively with different populations.^16^ As previously discussed, a multitude of vendors offers software for plaque and stenosis quantification.^26–29^^,^^35^ A comparative review of these tools is necessary to ensure consistency in plaque quantification across different platforms. Photon-counting CT and advanced reconstruction methods promise major improvements in spatial and spectral resolution, thereby enhancing plaque visualization and characterization.^63^^,^^67^ However, they will require the retraining and recalibration of existing AI algorithms, which up to now have mostly been trained on EID CCTA data.

Ultimately, the future of CCTA imaging will lie in its capacity to integrate all the aforementioned layers of information—plaque burden, composition, luminal stenosis, lesion-specific FFR, and plaque progression—alongside patient-specific factors, such as comorbidities, risk factors, and treatment response. In the future, the combined use of photon-counting detectors, AI-QCT, AI-CSQ, and FFR-CT will maximize the data extracted from CCTA, producing vast datasets from each scan. Emerging analytical capabilities have the potential to advance our understanding of the complex interplay between coronary anatomy and physiology, enabling more precise risk stratification and individualized management in patients with CAD.^93^ Future research should prioritize translating these multidimensional data into clinically actionable and pathophysiologically grounded insights to enhance diagnostic accuracy, therapeutic decision-making, and overall patient outcomes.

## Conclusion

Recent advances in noninvasive stenosis and plaque quantification, as well as FFR-CT and PCD-CT, have refined the radiological analysis of CAD, with important clinical implications. The benefits include improved imaging biomarkers, patient risk prognostication, follow-up treatment evaluation, and identification of patients requiring immediate revascularization. Future research should focus on the major obstacles to broad clinical implementation of these AI platforms, including the paucity of data on the robustness of AI-based evaluation, the reproducibility of reported metrics, and the need for standardized reference ranges for quantitative plaque metrics to guide patient management.

## References

[1] Klüner LV , Chan K, Antoniades C. Using artificial intelligence to study atherosclerosis from computed tomography imaging: a state-of-the-art review of the current literature. Atherosclerosis. 2024;398:117580. 10.1016/j.atherosclerosis.2024.117580 PMC11579307 38852022

[2] Schulze K , Stantien A-M, Williams MC, et al Coronary CT angiography evaluation with artificial intelligence for individualized medical treatment of atherosclerosis: a consensus statement from the QCI Study Group. Nat Rev Cardiol. 2025;23:100-115. 10.1038/s41569-025-01191-6 40751112

[3] Lin A , Manral N, Mcelhinney P, et al Deep learning-enabled coronary CT angiography for plaque and stenosis quantification and cardiac risk prediction: an international multicentre study. Lancet Digit Health. 2022;4:e256-e265. 10.1016/S2589-7500(22)00022-X PMC9047317 35337643

[4] Van Assen M , Von Knebel Doeberitz P, Quyyumi AA, De Cecco CN. Artificial intelligence for advanced analysis of coronary plaque. Eur Heart J Suppl. 2023;25:C112-C117. 10.1093/eurheartjsupp/suad038 PMC10132604 37125298

[5] Maurovich-Horvat P , Ferencik M, Voros S, Merkely B, Hoffmann U. Comprehensive plaque assessment by coronary CT angiography. Nat Rev Cardiol. 2014;11:390-402. 10.1038/nrcardio.2014.60 24755916

[6] Motoyama S , Sarai M, Harigaya H, et al Computed tomographic angiography characteristics of atherosclerotic plaques subsequently resulting in acute coronary syndrome. J Am Coll Cardiol. 2009;54:49-57. 10.1016/j.jacc.2009.02.068 19555840

[7] Ahmadi A , Argulian E, Leipsic J, Newby DE, Narula J. From subclinical atherosclerosis to plaque progression and acute coronary events. J Am Coll Cardiol. 2019;74:1608-1617. 10.1016/j.jacc.2019.08.012 31537271

[8] Williams MC , Kwiecinski J, Doris M, et al Low-attenuation noncalcified plaque on coronary computed tomography angiography predicts myocardial infarction: Results from the multicenter SCOT-HEART trial (Scottish Computed Tomography of the HEART). Circulation. 2020;141:1452-1462. 10.1161/CIRCULATIONAHA.119.044720 PMC7195857 32174130

[9] Williams MC , Moss AJ, Dweck M, et al Coronary artery plaque characteristics associated with adverse outcomes in the SCOT-HEART study. J Am Coll Cardiol. 2019;73:291-301. 10.1016/j.jacc.2018.10.066 PMC6342893 30678759

[10] Williams MC , Wereski R, Tuck C, et al; SCOT-HEART Investigators. Coronary CT angiography-guided management of patients with stable chest pain: 10-year outcomes from the SCOT-HEART randomised controlled trial in Scotland. Lancet. 2025;405:329-337. 10.1016/S0140-6736(24)02679-5 39863372

[11] Ferencik M , Mayrhofer T, Bittner DO, et al Use of high-risk coronary atherosclerotic plaque detection for risk stratification of patients with stable chest pain: a secondary analysis of the PROMISE randomized clinical trial. JAMA Cardiol. 2018;3:144-152. 10.1001/jamacardio.2017.4973 PMC5838601 29322167

[12] Van Rosendael AR , Bax AM, Smit JM, et al Clinical risk factors and atherosclerotic plaque extent to define risk for major events in patients without obstructive coronary artery disease: the long-term coronary computed tomography angiography CONFIRM registry. Eur Heart J Cardiovasc Imaging. 2020;21:479-488. 10.1093/ehjci/jez322 PMC7821703 32065624

[13] Nurmohamed NS , Min JK, Anthopolos R, et al Atherosclerosis quantification and cardiovascular risk: the ISCHEMIA trial. Eur Heart J. 2024;45:3735-3747. 10.1093/eurheartj/ehae471 PMC11439108 39101625

[14] Feuchtner G , Kerber J, Burghard P, et al The high-risk criteria low-attenuation plaque <60 HU and the napkin-ring sign are the most powerful predictors of MACE: a long-term follow-up study. Eur Heart J Cardiovasc Imaging. 2017;18:772-779. 10.1093/ehjci/jew167 27502292

[15] McDermott M , Meah MN, Khaing P, et al Rationale and design of SCOT-HEART 2 trial. JACC Cardiovasc Imaging. 2024;17:1101-1112. 10.1016/j.jcmg.2024.05.016 39001735

[16] Nieman K , García-García HM, Hideo-Kajita A, et al Standards for quantitative assessments by coronary computed tomography angiography (CCTA). J Cardiovasc Comput Tomogr. 2024;18:429-443. 10.1016/j.jcct.2024.05.232 PMC12782628 38849237

[17] Leipsic J , Abbara S, Achenbach S, et al SCCT guidelines for the interpretation and reporting of coronary CT angiography: a report of the Society of Cardiovascular Computed Tomography Guidelines Committee. J Cardiovasc Comput Tomogr. 2014;8:342-358. 10.1016/j.jcct.2014.07.003 25301040

[18] Narula J , Stuckey TD, Nakazawa G, et al Prospective deep learning–based quantitative assessment of coronary plaque by computed tomography angiography compared with intravascular ultrasound: the REVEALPLAQUE study. Eur Heart J Cardiovasc Imaging. 2024;25:1287-1295. 10.1093/ehjci/jeae115 PMC11346368 38700097

[19] Lu MT , Meyersohn NM, Mayrhofer T, et al Central core laboratory versus site interpretation of coronary CT angiography: agreement and association with cardiovascular events in the PROMISE trial. Radiology. 2018;287:87-95. 10.1148/radiol.2017172181 PMC5881637 29178815

[20] Budoff MJ , Dowe D, Jollis JG, et al Diagnostic performance of 64-Multidetector row coronary computed tomographic angiography for evaluation of coronary artery stenosis in individuals without known coronary artery disease. J Am Coll Cardiol. 2008;52:1724-1732. 10.1016/j.jacc.2008.07.031 19007693

[21] Shaw LJ , Blankstein R, Bax JJ, et al Society of cardiovascular computed tomography / North American society of cardiovascular imaging—expert consensus document on coronary CT imaging of atherosclerotic plaque. J Cardiovasc Comput Tomogr. 2021;15:93-109. 10.1016/j.jcct.2020.11.002 33303383

[22] Matsumoto H , Watanabe S, Kyo E, et al Standardized volumetric plaque quantification and characterization from coronary CT angiography: a head-to-head comparison with invasive intravascular ultrasound. Eur Radiol. 2019;29:6129-6139. 10.1007/s00330-019-06219-3 PMC6824266 31028446

[23] Dey D , Schepis T, Marwan M, Slomka PJ, Berman DS, Achenbach S. Automated three-dimensional quantification of noncalcified coronary plaque from coronary CT angiography: comparison with intravascular US. Radiology. 2010;257:516-522. 10.1148/radiol.10100681 20829536

[24] Van Rosendael AR , Lin FY, Ma X, et al Percent atheroma volume: optimal variable to report whole-heart atherosclerotic plaque burden with coronary CTA, the PARADIGM study. J Cardiovasc Comput Tomogr. 2020;14:400-406. 10.1016/j.jcct.2020.01.012 32063545

[25] Koo BK , Yang S, Jung JW, et al Artificial intelligence–enabled quantitative coronary plaque and hemodynamic analysis for predicting acute coronary syndrome. JACC Cardiovasc Imaging. 2024;17:1062-1076. 10.1016/j.jcmg.2024.03.015 38752951

[26] Ihdayhid AR , Tzimas G, Peterson K, et al Diagnostic performance of AI-enabled plaque quantification from coronary CT angiography compared with intravascular ultrasound. Radiol Cardiothorac Imaging. 2024;6:e230312. 10.1148/ryct.230312 PMC11683154 39540820

[27] Buckler AJ , Karlöf E, Lengquist M, et al Virtual transcriptomics: noninvasive phenotyping of atherosclerosis by decoding plaque biology from computed tomography angiography imaging. Arterioscler Thromb Vasc Biol. 2021;41:1738-1750. 10.1161/ATVBAHA.121.315969 PMC8062292 33691476

[28] Nurmohamed NS , Danad I, Jukema RA, et al; CREDENCE and PACIFIC-1 Investigators. Development and validation of a quantitative coronary CT angiography model for diagnosis of vessel-specific coronary ischemia. JACC Cardiovasc Imaging. 2024;17:894-906. 10.1016/j.jcmg.2024.01.007 38483420

[29] Omori H , Matsuo H, Fujimoto S, et al Determination of lipid-rich plaques by artificial intelligence-enabled quantitative computed tomography using near-infrared spectroscopy as reference. Atherosclerosis. 2023;386:117363. 10.1016/j.atherosclerosis.2023.117363 37944269

[30] Williams MC , Earls JP, Hecht H. Quantitative assessment of atherosclerotic plaque, recent progress and current limitations. J Cardiovasc Comput Tomogr. 2022;16:124-137. 10.1016/j.jcct.2021.07.001 34326003

[31] Chandrashekhar Y , Blankstein R, Shaw LJ, et al Quantitative coronary plaque analysis in clinical practice: 2025 ACC scientific statement. JACC Cardiovasc Imaging. 2025;19:637-652. 10.1016/j.jcmg.2025.11.008S1936878X25006540 41405512

[32] Versteylen MO , Kietselaer BL, Dagnelie PC, et al Additive value of semiautomated quantification of coronary artery disease using cardiac computed tomographic angiography to predict future acute coronary syndrome. J Am Coll Cardiol. 2013;61:2296-2305. 10.1016/j.jacc.2013.02.065 23562925

[33] Hell MM , Motwani M, Otaki Y, et al Quantitative global plaque characteristics from coronary computed tomography angiography for the prediction of future cardiac mortality during long-term follow-up. Eur Heart J Cardiovasc Imaging. 2017;18:1331-1339. 10.1093/ehjci/jex183 PMC5837249 28950315

[34] Van Rosendael AR , Maliakal G, Kolli KK, et al Maximization of the usage of coronary CTA derived plaque information using a machine learning based algorithm to improve risk stratification; insights from the CONFIRM registry. J Cardiovasc Comput Tomogr. 2018;12:204-209. 10.1016/j.jcct.2018.04.011 29753765

[35] Nurmohamed NS , Bom MJ, Jukema RA, et al AI-Guided quantitative plaque staging predicts long-term cardiovascular outcomes in patients at risk for atherosclerotic CVD. JACC Cardiovasc Imaging. 2024;17:269-280. 10.1016/j.jcmg.2023.05.020 37480907

[36] Saito Y , Kobayashi Y, Fujii K, et al CVIT 2025 clinical expert consensus document on intravascular ultrasound. Cardiovasc Interv and Ther. 2025;40:211-225. 10.1007/s12928-025-01090-0 PMC11910423 39870989

[37] Fujii K , Hao H, Shibuya M, et al Accuracy of OCT, grayscale IVUS, and their combination for the diagnosis of coronary TCFA. JACC Cardiovasc Imaging. 2015;8:451-460. 10.1016/j.jcmg.2014.10.015 25797121

[38] Brown AJ , Obaid DR, Costopoulos C, et al Direct comparison of virtual-histology intravascular ultrasound and optical coherence tomography imaging for identification of thin-cap fibroatheroma. Circ Cardiovasc Imaging. 2015;8:e003487. 10.1161/CIRCIMAGING.115.003487 PMC4596008 26429760

[39] Bell JS , Weir-McCall J, Nicol E, Lip GYH, Nørgaard BL, Fairbairn TA. Plaque quantification from coronary computed tomography angiography in predicting cardiovascular events: a systematic review and meta-analysis. J Cardiovasc Comput Tomogr. 2025;19:423-432. 10.1016/j.jcct.2025.05.003 40425410

[40] Miller RJH , Manral N, Lin A, et al Patient-Specific myocardial infarction risk thresholds from AI-enabled coronary plaque analysis. Circ Cardiovasc Imaging. 2024;17:e016958. 10.1161/CIRCIMAGING.124.016958 PMC11834154 39405390

[41] Dahdal J , Jukema RA, Maaniitty T, et al CCTA-Derived coronary plaque burden offers enhanced prognostic value over CAC scoring in suspected CAD patients. Eur Heart J Cardiovasc Imaging. 2025;26:945-954. 10.1093/ehjci/jeaf093 40131307

[42] American Medical Association. AMA Releases CPT 2026 Code Set. Published online September 11, 2025. Accessed October 21, 2025. https://www.ama-assn.org/press-center/ama-press-releases/ama-releases-cpt-2026-code-set

[43] Cury RC , Leipsic J, Abbara S, et al CAD-RADS^TM^ 2.0–2022 coronary artery disease-reporting and data system. JACC Cardiovasc Imaging. 2022;15:1974-2001. 10.1016/j.jcmg.2022.07.002 36115815

[44] Shrivastava P , Kashikar S, Parihar PH, Kasat P, Bhangale P, Shrivastava P. A systematic review on deep learning-enabled coronary CT angiography for plaque and stenosis quantification and cardiac risk prediction. Eur J Radiol Open. 2025;14:100652. 10.1016/j.ejro.2025.100652 PMC12123344 40453037

[45] Van Noort D , Guo L, Leng S, et al Evaluating machine learning accuracy in detecting significant coronary stenosis using CCTA-derived fractional flow reserve: Meta-analysis and systematic review. Int J Cardiol Heart Vasc. 2024;55:101528. 10.1016/j.ijcha.2024.101528 PMC11795686 39911616

[46] Bernardo R , Nurmohamed NS, Bom MJ, et al Diagnostic accuracy in coronary CT angiography analysis: artificial intelligence versus human assessment. Open Heart. 2025;12:e003115. 10.1136/openhrt-2024-003115 PMC11784206 39800437

[47] Dundas J , Leipsic JA, Sellers S, et al Artificial intelligence–based coronary stenosis quantification at coronary CT angiography versus quantitative coronary angiography. Radiology: Cardiothoracic Imaging. 2023;5:e230124. 10.1148/ryct.230124 PMC11163244 38166336

[48] Farì R , Van Assen M, Quintana R, et al Impact of deep learning–based artificial intelligence assistance on reader agreement in coronary CT angiography interpretation. Radiology: Cardiothoracic Imaging. 2025;7:e240563. 10.1148/ryct.240563 41065550

[49] Narula J , Chandrashekhar Y, Ahmadi A, et al SCCT 2021 expert consensus document on coronary computed tomographic angiography: a report of the society of cardiovascular computed tomography. J Cardiovasc Comput Tomogr. 2021;15:192-217. 10.1016/j.jcct.2020.11.001 PMC8713482 33303384

[50] Arnett DK , Blumenthal RS, Albert MA, et al 2019 ACC/AHA guideline on the primary prevention of cardiovascular disease: a report of the American college of cardiology/American heart association task force on clinical practice guidelines. Circulation. 2019;140:e596-e646. 10.1161/CIR.0000000000000678 PMC7734661 30879355

[51] Mach F , Baigent C, Catapano AL, et al; ESC Scientific Document Group. 2019 ESC/EAS guidelines for the management of dyslipidaemias: lipid modification to reduce cardiovascular risk. Eur Heart J. 2020;41:111-188. 10.1093/eurheartj/ehz455 31504418

[52] Visseren FLJ , Mach F, Smulders YM, et al; ESC Scientific Document Group. 2021 ESC guidelines on cardiovascular disease prevention in clinical practice. Eur Heart J. 2021;42:3227-3337. 10.1093/eurheartj/ehab484 34458905

[53] Grandhi GR , Mirbolouk M, Dardari ZA, et al Interplay of coronary artery calcium and risk factors for predicting CVD/CHD mortality. JACC Cardiovasc Imaging. 2020;13:1175-1186. 10.1016/j.jcmg.2019.08.024 31734198

[54] Tzimas G , Gulsin GS, Everett RJ, et al Age- and sex-specific nomographic CT quantitative plaque data from a large international cohort. JACC Cardiovasc Imaging. 2024;17:165-175. 10.1016/j.jcmg.2023.05.011 37410009

[55] Slipczuk L , Blankstein R, Bucciarelli-Ducci C, et al; American Heart Association Cardiac Imaging and Intervention Committee of the Council on Clinical Cardiology and Council on Cardiovascular Radiology and Intervention; Council on Cardiovascular and Stroke Nursing; and Council on Quality of Care and Outcomes Research State of the art: evaluation and medical management of nonobstructive coronary artery disease in patients with chest pain: a scientific statement from the American Heart Association. Circulation. 2025;152:e443-e466. 10.1161/CIR.0000000000001394 41208705

[56] Banach M , Serban C, Sahebkar A, et al; Lipid and Blood Pressure Meta-Analysis Collaboration (LBPMC) Group. Impact of statin therapy on coronary plaque composition: a systematic review and meta-analysis of virtual histology intravascular ultrasound studies. BMC Med. 2015;13:229. 10.1186/s12916-015-0459-4

[57] Rivera FB , Cha SW, Varona MC, et al Atherosclerotic coronary plaque regression from lipid-lowering therapies: a meta-analysis and meta-regression. Am J Prev Cardiol. 2024;18:100645. 10.1016/j.ajpc.2024.100645 PMC10966153 38550634

[58] Oesterle A , Laufs U, Liao JK. Pleiotropic effects of statins on the cardiovascular system. Circ Res. 2017;120:229-243. 10.1161/CIRCRESAHA.116.308537 PMC5467317 28057795

[59] Budoff MJ , Muhlestein JB, Bhatt DL, et al Effect of icosapent ethyl on progression of coronary atherosclerosis in patients with elevated triglycerides on statin therapy: a prospective, placebo-controlled randomized trial (EVAPORATE): interim results. Cardiovasc Res. 2021;117:1070-1077. 10.1093/cvr/cvaa184 32609331

[60] Nicholls SJ , Puri R, Anderson T, et al Effect of evolocumab on coronary plaque composition. J Am Coll Cardiol. 2018;72:2012-2021. 10.1016/j.jacc.2018.06.078 30336824

[61] McDermott M , Khaing PH, Meah MN, et al CT angiography, healthy lifestyle behaviors, and preventive therapy: a nested substudy of the SCOT-HEART 2 randomized clinical trial. JAMA Cardiol. 2025;10:841-850. 10.1001/jamacardio.2025.1763 PMC12177732 40531507

[62] Andelius L , Mortensen MB, Nørgaard BL, Abdulla J. Impact of statin therapy on coronary plaque burden and composition assessed by coronary computed tomographic angiography: a systematic review and meta-analysis. Eur Heart J Cardiovasc Imaging. 2018;19:850-858. 10.1093/ehjci/jey012 29617981

[63] Flohr T , Schmidt B, Ulzheimer S, Alkadhi H. Cardiac imaging with photon counting CT. Br J Radiol. 2023;96:20230407. 10.1259/bjr.20230407 PMC10646663 37750856

[64] Williams MC , Newby DE. Photon-counting CT: a step change leading to a revolution in coronary imaging. Radiology. 2023;307:e231234. 10.1148/radiol.231234 PMC10315513 37338352

[65] Laux GS , Halfmann MC, Kavermann L, et al Ultra-high resolution photon-counting detector coronary CT minimizes overestimation bias compared to invasive reference. Eur J Radiol. 2025;188:112154. 10.1016/j.ejrad.2025.112154 40347824

[66] Vecsey-Nagy M , Tremamunno G, Schoepf UJ, et al Coronary plaque quantification with ultrahigh-spatial-resolution photon-counting detector CT: intraindividual comparison with energy-integrating detector CT. Radiology. 2025;314:e241479. 10.1148/radiol.241479 40035676

[67] Shiyovich A , Singh A, Blair CV, et al Photon-counting computed tomography in cardiac imaging. JACC Cardiovasc Imaging. 2025;19:94-117. 10.1016/j.jcmg.2025.07.022S1936878X25004759 41065616

[68] Meijboom WB , Van Mieghem CAG, Van Pelt N, et al Comprehensive assessment of coronary artery stenoses. J Am Coll Cardiol. 2008;52:636-643. 10.1016/j.jacc.2008.05.024 18702967

[69] De Bruyne B , Pijls NHJ, Heyndrickx GR, Hodeige D, Kirkeeide R, Gould KL. Pressure-derived fractional flow reserve to assess serial epicardial stenoses: theoretical basis and animal validation. Circulation. 2000;101:1840-1847. 10.1161/01.CIR.101.15.1840 10769286

[70] Zimmermann FM , Ferrara A, Johnson NP, et al Deferral vs. performance of percutaneous coronary intervention of functionally non-significant coronary stenosis: 15-year follow-up of the DEFER trial. Eur Heart J. 2015;36:3182-3188. 10.1093/eurheartj/ehv452 26400825

[71] Tonino PAL , Fearon WF, De Bruyne B, et al Angiographic versus functional severity of coronary artery stenoses in the FAME study. J Am Coll Cardiol. 2010;55:2816-2821. 10.1016/j.jacc.2009.11.096 20579537

[72] Farhad A , Reza R, Azamossadat H, et al Artificial intelligence in estimating fractional flow reserve: a systematic literature review of techniques. BMC Cardiovasc Disord. 2023;23:407. 10.1186/s12872-023-03447-w PMC10439535 37596521

[73] Sharma P , Itu L, Zheng X, et al A framework for personalization of coronary flow computations during rest and hyperemia. In: *2012 Annual International Conference of the IEEE Engineering in Medicine and Biology Society*. IEEE; 2012:6665-6668. 10.1109/EMBC.2012.6347523 23367458

[74] Koo BK , Erglis A, Doh JH, et al Diagnosis of ischemia-causing coronary stenoses by noninvasive fractional flow reserve computed from coronary computed tomographic angiograms. J Am Coll Cardiol. 2011;58:1989-1997. 10.1016/j.jacc.2011.06.066 22032711

[75] Min JK , Leipsic J, Pencina MJ, et al Diagnostic accuracy of fractional flow reserve from anatomic CT angiography. JAMA. 2012;308:1237-1245. 10.1001/2012.jama.11274 PMC4281479 22922562

[76] Nørgaard BL , Leipsic J, Gaur S, et al; NXT Trial Study Group. Diagnostic performance of noninvasive fractional flow reserve derived from coronary computed tomography angiography in suspected coronary artery disease. J Am Coll Cardiol. 2014;63:1145-1155. 10.1016/j.jacc.2013.11.043 24486266

[77] Douglas PS , Pontone G, Hlatky MA, et al; PLATFORM Investigators. Clinical outcomes of fractional flow reserve by computed tomographic angiography-guided diagnostic strategies vs. usual care in patients with suspected coronary artery disease: the prospective longitudinal trial of FFR_CT_ : outcome and resource impacts study. Eur Heart J. 2015;36:3359-3367. 10.1093/eurheartj/ehv444 PMC4677273 26330417

[78] Driessen RS , Danad I, Stuijfzand WJ, et al Comparison of coronary computed tomography angiography, fractional flow reserve, and perfusion imaging for ischemia diagnosis. J Am Coll Cardiol. 2019;73:161-173. 10.1016/j.jacc.2018.10.056 30654888

[79] Douglas PS , Nanna MG, Kelsey MD, et al; PRECISE Investigators. Comparison of an initial Risk-Based testing strategy vs usual testing in stable symptomatic patients with suspected coronary artery disease: the PRECISE randomized clinical trial. JAMA Cardiol. 2023;8:904-914. 10.1001/jamacardio.2023.2595 PMC10448364 37610731

[80] Curzen N , Nicholas Z, Stuart B, et al Fractional flow reserve derived from computed tomography coronary angiography in the assessment and management of stable chest pain: the FORECAST randomized trial. Eur Heart J. 2021;42:3844-3852. 10.1093/eurheartj/ehab444 PMC8648068 34269376

[81] Fairbairn TA , Nieman K, Akasaka T, et al Real-world clinical utility and impact on clinical decision-making of coronary computed tomography angiography-derived fractional flow reserve: lessons from the ADVANCE registry. Eur Heart J. 2018;39:3701-3711. 10.1093/eurheartj/ehy530 PMC6215963 30165613

[82] Nørgaard BL , Gaur S, Fairbairn TA, et al Prognostic value of coronary computed tomography angiographic derived fractional flow reserve: a systematic review and meta-analysis. Heart. 2022;108:194-202. 10.1136/heartjnl-2021-319773 PMC8762006 34686567

[83] Madsen KT , Nørgaard BL, Øvrehus KA, et al Prognostic value of coronary CT angiography–derived fractional flow reserve on 3-year outcomes in patients with stable angina. Radiology. 2023;308:e230524. 10.1148/radiol.230524 37698477

[84] Gulati M , Levy PD, Mukherjee D, et al 2021 AHA/ACC/ASE/CHEST/SAEM/SCCT/SCMR guideline for the evaluation and diagnosis of chest pain: a report of the American College of Cardiology/American Heart Association Joint Committee on clinical practice guidelines. Circulation. 2021;144:e368-e454. 10.1161/CIR.0000000000001029 34709879

[85] Fairbairn TA , Mullen L, Nicol E, et al Implementation of a national AI technology program on cardiovascular outcomes and the health system. Nat Med. 2025;31:1903-1910. 10.1038/s41591-025-03620-y PMC12176617 40186078

[86] Coenen A , Kim YH, Kruk M, et al Diagnostic accuracy of a machine-learning approach to coronary computed tomographic angiography–based fractional flow reserve: result from the MACHINE Consortium. Circ Cardiovasc Imaging. 2018;11:e007217. 10.1161/CIRCIMAGING.117.007217 29914866

[87] Yang J , Shan D, Wang X, et al On-Site computed tomography–derived fractional flow reserve to guide management of patients with stable coronary artery disease: the TARGET randomized trial. Circulation. 2023;147:1369-1381. 10.1161/CIRCULATIONAHA.123.063996 36870065

[88] Rabbat MG , Berman DS, Kern M, et al Interpreting results of coronary computed tomography angiography-derived fractional flow reserve in clinical practice. J Cardiovasc Comput Tomogr. 2017;11:383-388. 10.1016/j.jcct.2017.06.002 28666784

[89] Takagi H , Leipsic JA, McNamara N, et al Trans-lesional fractional flow reserve gradient as derived from coronary CT improves patient management: ADVANCE registry. J Cardiovasc Comput Tomogr. 2022;16:19-26. 10.1016/j.jcct.2021.08.003 PMC9719736 34518113

[90] Yan H , Gao Y, Zhao N, et al Change in computed tomography-derived fractional flow reserve across the lesion improve the diagnostic performance of functional coronary stenosis. Front Cardiovasc Med. 2022;8:788703. 10.3389/fcvm.2021.788703 PMC8792740 35097009

[91] Lee SH , Hong D, Dai N, et al Anatomic and hemodynamic plaque characteristics for subsequent coronary events. Front Cardiovasc Med. 2022;9:871450. 10.3389/fcvm.2022.871450 PMC9167998 35677691

[92] Cai Z , Yu T, Yang Z, et al Detecting lesion-specific ischemia in patients with coronary artery disease with computed tomography fractional flow reserve measured at different sites. BMC Med Imaging. 2023;23:76. 10.1186/s12880-023-01031-4 PMC10242782 37277697

[93] Leipsic JA , Tzimas G. CT-derived plaque physiology and characterization. JACC Cardiovasc Imaging. 2024;17:298-300. 10.1016/j.jcmg.2023.08.018 37855800

[94] Wu J , Yang D, Zhang Y, et al Non-invasive imaging innovation: FFR-CT combined with plaque characterization, safeguarding your cardiac health. J Cardiovasc Comput Tomogr. 2025;19:152-158. 10.1016/j.jcct.2024.08.008 39299900

[95] Gaur S , Øvrehus KA, Dey D, et al Coronary plaque quantification and fractional flow reserve by coronary computed tomography angiography identify ischaemia-causing lesions. Eur Heart J. 2016;37:1220-1227. 10.1093/eurheartj/ehv690 PMC4830909 26763790

[96] Von Knebel Doeberitz PL , De Cecco CN, Schoepf UJ, et al Impact of coronary computerized tomography angiography-derived plaque quantification and machine-learning computerized tomography fractional flow reserve on adverse cardiac outcome. Am J Cardiol. 2019;124:1340-1348. 10.1016/j.amjcard.2019.07.061 31481177

[97] Dundas J , Leipsic J, Fairbairn T, et al Interaction of AI-enabled quantitative coronary plaque volumes on coronary CT angiography, FFR_CT_, and clinical outcomes: a retrospective analysis of the ADVANCE registry. Circ: Cardiovascular Imaging. 2024;17:e016143. 10.1161/CIRCIMAGING.123.016143 38469689

[98] Hakim D , Coskun AU, Maynard C, et al Endothelial shear stress computed from coronary computed tomography angiography: a direct comparison to intravascular ultrasound. J Cardiovasc Comput Tomogr. 2023;17:201-210. 10.1016/j.jcct.2023.03.009 PMC10993230 37076326

[99] Yang S , Jung JW, Park SH, et al Prognostic time frame of plaque and hemodynamic characteristics and integrative risk prediction for acute coronary syndrome. JACC Cardiovasc Imaging. 2025;18:784-795. 10.1016/j.jcmg.2025.02.003 40272335

[100] Choi G , Lee JM, Kim HJ, et al Coronary artery axial plaque stress and its relationship with lesion geometry. JACC Cardiovasc Imaging. 2015;8:1156-1166. 10.1016/j.jcmg.2015.04.024 26363834

[101] Rabbat M , Leipsic J, Bax J, et al Fractional flow reserve derived from coronary computed tomography angiography safely defers invasive coronary angiography in patients with stable coronary artery disease. J Clin Med. 2020;9:604. 10.3390/jcm9020604 PMC7074264 32102371

[102] Tanisawa H , Matsumoto H, Cadet S, et al Quantification of low-attenuation plaque burden from coronary CT angiography: a head-to-head comparison with near-infrared spectroscopy intravascular US. Radiol Cardiothorac Imaging. 2023;5:e230090. 10.1148/ryct.230090 PMC10613924 37908555

[103] Razavi AC , Agatston AS, Shaw LJ, et al Evolving role of calcium density in coronary artery calcium scoring and atherosclerotic cardiovascular disease risk. JACC Cardiovasc Imaging. 2022;15:1648-1662. 10.1016/j.jcmg.2022.02.026 PMC9908416 35861969

[104] Van Rosendael AR , Narula J, Lin FY, et al Association of high-density calcified 1K plaque with risk of acute coronary syndrome. JAMA Cardiol. 2020;5:282-290. 10.1001/jamacardio.2019.5315 PMC6990946 31968065

